# CX_3_CL1 Recruits NK Cells Into the Central Nervous System and Aggravates Brain Injury of Mice Caused by *Angiostrongylus cantonensis* Infection

**DOI:** 10.3389/fcimb.2021.672720

**Published:** 2021-05-04

**Authors:** Rong Zhang, Tingting Miao, Min Qin, Chengsi Zhao, Wei Wang, Chengcheng Zhang, Xinjian Liu, Ying Chen, Ailing Chen, Yong Wang

**Affiliations:** ^1^ Experimental Teaching Center of Basic Medicine, Nanjing Medical University, Nanjing, China; ^2^ Department of Pathogen Biology, Key Laboratory of Pathogen Biology of Jiangsu Province, Nanjing Medical University, Nanjing, China; ^3^ Translational Medicine Laboratory, Research Institute for Reproductive Health and Genetic Diseases, The Affiliated Wuxi Maternity and Child Health Care Hospital of Nanjing Medical University, Wuxi, China

**Keywords:** CX_3_CL1, NK cells, *Angiostrongylus cantonensis*, infection, brain injury, central nervous system

## Abstract

**Background:**

*Angiostrongylus cantonensis* (*A. cantonensis*), is a food-borne zoonotic parasite that can cause central nervous system (CNS) injury characterized by eosinophilic meningitis. However, the pathogenesis of angiostrongylosis remains elusive. Natural killer cells (NK cells) are unique innate lymphocytes important in early defense against pathogens. The aim of this study was to investigate the role of NK cells in *A. cantonensis* infection and to elucidate the key factors that recruit NK cells into the CNS.

**Methods:**

Mouse model of *A. cantonensis* infection was established by intragastric administration of third-stage larvae. The expression of cytokines and chemokines at gene and protein levels was analyzed by qRT-PCR and ELISA. Distribution of NK cells was observed by immunohistochemistry and flow cytometry. NK cell-mediated cytotoxicity against YAC-1 cells was detected by LDH release assay. The ability of NK cells to secrete cytokines was determined by intracellular flow cytometry and ELISA. Depletion and adoptive transfer of NK cells *in vivo* was induced by tail vein injection of anti-asialo GM1 rabbit serum and purified splenic NK cells, respectively. CX_3_CL1 neutralization experiment was performed by intraperitoneal injection of anti-CX_3_CL1 rat IgG.

**Results:**

The infiltration of NK cells in the CNS of *A. cantonensis*-infected mice was observed from 14 dpi and reached the peak on 18 and 22 dpi. Compared with uninfected splenic NK cells, the CNS-infiltrated NK cells of infected mice showed enhanced cytotoxicity and increased IFN-γ and TNF-α production ability. Depletion of NK cells alleviated brain injury, whereas adoptive transfer of NK cells exacerbated brain damage in *A. cantonensis*-infected mice. The expression of CX_3_CL1 in the brain tissue and its receptor CX_3_CR1 on the CNS-infiltrated NK cells were both elevated after *A. cantonensis* infection. CX_3_CL1 neutralization reduced the percentage and absolute number of the CNS-infiltrated NK cells and relieved brain damage caused by *A. cantonensis* infection.

**Conclusions:**

Our results demonstrate that the up-regulated CX_3_CL1 in the brain tissue recruits NK cells into the CNS and aggravates brain damage caused by *A. cantonensis* infection. The findings improve the understanding of the pathogenesis of angiostrongyliasis and expand the therapeutic intervention in CNS disease.

## Introduction

*Angiostrongylus cantonensis* (*A. cantonensis*), is a food-borne zoonotic parasite that can cause damage to the central nervous system (CNS) (Wang et al., 2012). With the development of global logistics transportation and climate warming, *A. cantonensis* has spread from its traditional endemic regions of Southeast Asia and the Pacific islands to the American continent, Europe, Africa and Australia (Gelis et al., 2011; Iwanowicz et al., 2015; Liu et al., 2018; Rael et al., 2018; Barbosa et al., 2020; Federspiel et al., 2020). Angiostrongyliasis has become a potentially fatal globally emerging infectious disease.

Humans and mouse are both non-permissive hosts of *A. cantonensis* and become infected *via* ingestion of raw or undercooked intermediate hosts including snails or slugs, or vegetables or water contaminated by the infective third-stage larvae (Barratt et al., 2016). After penetrating the intestinal wall, the larvae migrate in the body with the flow of blood, and finally settle in the CNS. These larvae in non-permissive hosts cannot develop into adults, but can survive in the form of larvae for a long time (Lv et al., 2017). Most patients when infected with *A. cantonensis* develop eosinophilic meningitis and common clinical symptoms include headache, fever, neck stiffness, paresthesia and vomiting (Martins et al., 2015). However, the pathogenesis of angiostrongyliasis is not fully understood. Mechanical damage to the CNS caused by the larvae’ movements, inflammation and immune response induced by the larval secretion and excreta, may be involved (Gosnell and Kramer, 2013; Martins et al, 2015; Mengying et al., 2017).

Natural killer cells (NK cells) are a special type of lymphocyte critical to the innate immune system. They are able to rapidly kill target cells by cytotoxicity without antigen presentation and regulate immune response by secreting various cytokines and chemokines (Vivier et al., 2011). NK cells are one of the earliest cell types to arrive at target organs of inflammation. It was reported that NK cells could migrate to the CNS under several pathological conditions, such as brain ischemia, traumatic injury, or infections (Hao et al., 2010; Gan et al., 2014; Zhang et al., 2014; Li et al., 2020). However, very little is known about whether NK cells are involved in the brain injury caused by *A. cantonensis* infection. NK cells derived from bone marrow and migrate through the blood to the spleen, liver, lung and many other organs (Abel et al., 2018). They can respond to a large array of chemokines and be recruited to distinct sites during physiological and pathological conditions. For example, CCL2 attracts CCR2^+^ NK cells to the liver during murine cytomegalovirus (MCMV) infection (Hokeness et al., 2005), CX_3_CL1 recruit CX_3_CR1^+^ NK cells into the CNS of experimental autoimmune encephalomyelitis (EAE) mice (Huang et al., 2006), CXCL10 attracts CXCR3^+^ NK cells accumulate into the ischemic brain tissues (Zhang et al., 2014), CCL3/CCL4/CCL5 attracts CCR5^+^ NK cells to the synovial fluid of rheumatoid arthritis (RA) patients (Parolini et al., 2007). The mechanism of recruitment of NK cells across the blood-brain barrier (BBB) into the CNS needs to be studied.

The aim of this study was to investigate the role of NK cells in the brain injury caused by *A. cantonensis* infection and to elucidate the key factors that recruit NK cells into the CNS. Our findings will be helpful to further understand the pathogenesis of angiostrongyliasis and expand therapeutic intervention in CNS disease.

## Materials and Methods

### Animal Experiments

A total of 300 female BALB/c mice (6–8 weeks old and weighing 18-20 g) were purchased from the Animal Core Facility of Nanjing Medical University (China), maintained in a specific pathogen-free environment, and provided unlimited access to food and water. All experiments were performed in strict compliance with the institutional guidelines and were approved by the Institutional Animal Care and Use Committee of Nanjing Medical University (Approval No. IACUC-1812040). Each mouse was infected with 20 *A. cantonensis* third-stage larvae (L3) by intragastric administration. L3 were isolated from *A. cantonensis*-infected Biomphalaria glabrata using the method described previously (Mengying et al., 2017). The mice were euthanized on 10, 14, 18, 22, and 26 days post-infection (dpi).

### Experimental Grouping

The experiment was grouped according to different objectives.

To detect the effect of *A. cantonensis* infection on mice and the percentage and absolute number of NK cells, the mice were divided into five groups according to the time of infection with twelve mice per group: 0, 10, 14, 18 and 22 dpi.

When detecting the phenotypic and functional changes of NK cells, cells were isolated from mice on 18 dpi and divided into three groups with three to four mice per group: splenic NK cells of uninfected mice (uninfected sNK), splenic NK cells of infected mice (infected sNK) and brain NK cells of infected mice (infected bNK).

In the experiment of NK cell depletion, NK cell adoptive transfer and CX_3_CL1 neutralization, the mice were euthanatized on 18 dpi and divided into four groups with twelve mice per group: uninfected group, infected group, NK depleted/NK transferred/CX_3_CL1 neutralized group and depleted/transferred/isotype control group.

### Neurological Impairment Evaluation

Longa’s score, Clark’s general score and Clark’s focal score were used to evaluate the neurological impairment of mice infected with *A. cantonensis*. Longa’s score is based on a five-point scale, where 0 point indicates no neurologic deficit, 1 point (failure to extend forepaw fully) a mild focal neurologic deficit, 2 point (circling to one side) a moderate focal neurologic deficit, 3 point (falling to one side) a severe focal deficit, and animals with 4 point cannot walk spontaneously or lose consciousness (Clark et al., 1997). Clark’s general score includes hair, ears, eyes, posture, autonomous movement, and epileptic seizures. Clark’s focal score comprises of body symmetry, gait, climbing, rotation test, forelimb symmetry and beard reactivity (Longa et al., 1989). Clark’s general score and Clark’s focal score are between 0 and 28. The higher the score, the more serious the neurological impairment is. The mice were scored by two technicians using a blind method.

### Histopathological Examination

Mice were perfused transcardially with 0.9% sodium chloride followed by 4% paraformaldehyde after anesthetized with 2% pentobarbital sodium (Sigma-Aldrich, USA). Brain samples were collected, fixed in 10% neutral formalin, embedded in paraffin, and cut into 3 μm-thick sections. Brain sections were then de-paraffinized in xylene, rehydrated *via* graded alcohols and stained with hematoxylin and eosin (H&E) (Biosharp, Wuhan, China). The sections were observed and photographed under a light microscope (Leica, Heidelberg, Germany).

For immunohistochemistry (IHC) analysis, brain sections were subjected to antigen retrieval by boiling the slices in citrate buffer (pH 6.0) with high heat for 15 min. Then sections were treated with 3% H_2_O_2_ for 10 min to remove endogenous peroxidase, blocked with 5% rabbit serum at room temperature for 20 min, and incubated with rabbit anti-mouse CD49b monoclonal antibody (mAb) (Abcam, Cambridge, UK) at 4°C overnight. After being washed in PBS, the sections were incubated with an HRP-conjugated secondary antibody (DAKO, Glostrup, Denmark) at room temperature for 15 min and then stained with 3, 3’-diaminobenzidine (DAB) for 10 min. Haematoxylin was used for cell nuclei detection. The sections were visualized and digitally scanned with a light microscope.

### Quantitative Reverse Transcription PCR (qRT-PCR)

Total RNA was extracted from mouse brains and NK cells using TRIzol Reagent (Thermo Fisher Scientific, USA) and reverse-transcribed to cDNA using a PimerScript™ RT Master Mix (TaKaRa, Kusatsu, Japan). qRT-PCR was performed on the LightCycler480^®^ Real-Time PCR System (Roche, Reinach, Switzerland) with the RealUniversal Color PreMix (SYBR Green) (Tiangen, Beijing, China), in accordance with the manufacturer’s instructions. The primer sequences were shown in **Table S1**. The mRNA levels of these genes were measured by the Ct value (threshold cycle), and the relative expression levels were calculated with the 2^-ΔΔCt^ method.

### Detection of Cytokine Expression in Brain Tissue

Each brain tissue was added into 4 mL tissue lysate (RayBiotech, USA) and 20 μL protease inhibitor (Merck, Germany) and homogenized in a gentle MACS separator (MiltenyiBiotec, Bergisch Gladbach, Germany). The total protein concentration in each sample was detected by BCA Protein Assay Kit (Tiangen, Beijing, China) according to the manufacturer’s instructions. And then, the levels of cytokines (IL-1β, IL-6 and TNF-α) and chemokines (CCL1, CCL2, CCL3, CCL4, CCL5, CXCL10 and CX_3_CL1 in brain tissue homogenate samples were determined with commercial ELISA Kits (MultiSciences, Hangzhou, China) according to the manufacturer’s manuals.

### Cell Isolation

Blood was collected by eyeball bleeding and gathered in tubes with 1% heparin sodium (Sigma-Aldrich, USA). The mice were anesthetized and perfused as described previously. And then, the brain, spleen, tibia, and femur were collected from the mice respectively.

Brain tissues were homogenized in grinders and filtered through a 70-μm cell strainer. And then cell pellets were resuspended in 30% Percoll (GE Healthcare, Pittsburgh, USA) and centrifuged against 70% Percoll. The cells between the 30–70% Percoll interfaces were collected as the brain mononuclear cells. Spleen was grinded with a syringe core and filtered through a nylon membrane. Erythrocytes were lysed and removed using Red Cell Lysis Buffer (Beyotime, Shanghai, China). Blood was double diluted and layered on the Ficoll-Paque (GE Healthcare, Pittsburgh, USA). After centrifugation, peripheral blood mononuclear cells (PBMCs) were distributed between plasma and Ficoll-Paque. PBMCs were transferred and resuspended in PBS. The tibia and femur bones were used to prepare bone marrow cells. The medullary cavity was washed repeatedly with RPMI-1640 (Gibco B, Gaithersburg, MD, USA) by syringe and bone marrow cells were collected and separated from erythrocytes.

NK cells were purified from brain mononuclear cells and splenic lymphocytes using a magnetic cell sorting system (MACS) incorporating anti-mice CD49 MicroBeads (MiltenyiBiotec, Bergisch Gladbach, Germany), following the manufacturer’s instructions. The purity of CD3^-^CD49^+^ NK cells after sorting was over 90% detected by FCM (**Figure S1**).

### Flow Cytometry (FCM)

Cells isolated from the brain, spleen, peripheral blood, and bone marrow were prepared to single cell suspension and resuspended in FCM buffer (0.5% BSA in PBS). For Cell surface marker detection, cells were incubated with TruStain FcX™ anti- CD16/32 (Biolegend, San Diego, USA) to block Fc-receptor for 5 min at 4 °C and then stained with the following specific antibodies: anti-CD45-percp-cy5.5, anti-CD3-FITC, anti-CD49b-APC, anti-CD122-PE, anti-CD69-PE, anti-NKp46-PE, anti-NKG2D-PE, anti-NKG2A-PE, anti-CD107a-PE mAbs or isotype controls (Biolegend, San Diego, USA) for 30 min at 4 °C. Cells were then detected on a Verse flow cytometer (BD Biosciences, San Jose, CA, USA). Data analysis was performed using FlowJo software (TreeStar, Ashland, USA). Gating strategy for the mouse CD45^+^CD3^-^CD49b^+^ NK cell population was shown in **Figure S2**.

For intracellular cytokine analysis, cells were cultured at a density of 2 × 10^6^/ml densities in 12-well plates and stimulated with 2 μL/mL Leukocyte Activation Cocktail plus GolgiPlug (BD Biosciences, San Jose, CA, USA) for 5 h. Cells were collected and stained with anti-CD45-percp-cy5.5, anti-CD3-FITC, anti-CD49b-APC mAbs for 30 min at 4 °C. After washing, cells were fixed and permeabilized using Cytofix/Cytoperm™ Fixation/Permeabilization Kit (BD Biosciences, San Jose, CA, USA) according to the manufacturer’s instructions. And then cells were incubated with anti-TNF-α-PE, anti- IFN-γ-BV421 mAbs or isotype controls (Biolegend, San Diego, USA) for 30 min at 4 °C. Cells were detected and data were analyzed as described previously.

### NK Cell Cytotoxicity Assays

NK cell-mediated cytotoxicity was determined using the Cytotoxicity Detection Kit^PLUS^ (Roche, Reinach, Switzerland) based on the measurement of LDH released from damaged cells according to the manufacturer’s manual. Purified NK cells (as effector cells) were incubated with YAC-1 cells (as target cells) at various effector cell/target cell ratios (1:1, 5:1, 10:1, 20:1) in 96-well plates for 3.5 h. All test samples were prepared in triplicate. Reaction mixture and stop solution were added into each well in turn. The absorbance of the samples was measured at 490 nm by an ELISA reader. The percentage of NK cell-mediated cytotoxicity was calculated by ODs using the following equation: Cytotoxicity (%) = (effector/target cell mix - effector cell control - low control)/(high control - low control) × 100.

### Detection of NK Cells Cytokine Secretion

Freshly purified NK cells were cultured in 24-well plates at a density of 1 × 10^6^/mL in the presence of 1 ng/mL IL-12 (PeproTech, Rocky Hill, NJ, USA). After 24 h of incubation, the culture supernatants were harvested. The concentrations of TNF-α and IFN- γ in supernatants were detected using Mouse TNF-α or IFN- γ High Sensitivity ELISA Kits (MultiSciences, Hangzhou, China) according to the instructions of the manufacturer.

### Depletion of NK Cells

5-7 Ganglio-N-tetraosylceramide (asialo GM1) is a glycolipid expressed on NK cells in mice, rats, and humans. Depletion of NK cells *in vivo* in infected mice was induced by tail vein injection of anti-asialo GM1 rabbit serum (Wako Pure Chemical Industries, Japan) according to the instructions from manufacturer and other researchers (Nishikado et al., 2011; Golic et al., 2016). Injection time and dose were shown in **Table 1**. Normal rabbit serum (Abbkine, CA, USA) were given to the infected mice at the same time as control. To evaluate the effect of NK cell depletion, the mice were euthanatized at 18 dpi and the percentage and absolute number of NK cells in brain and spleen were analyzed by FCM.

**Table 1:** Injection time and dose of anti-asialo GM1.

Injection	1st	2nd	3rd	4th
Days post-infection	0	5	10	15
Dose (μL per mouse)	20	20	20	20

### Adoptive Transfer of NK Cells

The splenic NK cells from normal mice were purified using MACS and were suspended in PBS. The NK cells (1 × 10^6^/mouse in 200 μL PBS) were transferred to the infected mice by tail vein injection on 12 dpi according to the protocols published by other investigators (Voynova et al., 2015). The respective diluents were injected to the control mice simultaneously. To evaluate the effect of adoptive transfer, the mice were euthanatized at 18 dpi and the percentage and absolute number of NK cells in brain and spleen were analyzed by FCM.

### *In Vivo* CX_3_CL1 Neutralization Experiment

To neutralize CX_3_CL1 *in vivo*, *A. cantonensis-*infected mice were injected intraperitoneally with anti-CX_3_CL1 rat IgG (R&D Systems, MN, USA) or isotype anti-rat IgG (R&D Systems, MN, USA) (4 μg/mouse) once a day from 10 dpi to 17 dpi according to the instructions from manufacturer and other researchers (Mills et al., 2012; Okuma et al., 2017). The mice were euthanatized and detected at 18 dpi to evaluate the effect of CX_3_CL1 neutralization.

### Statistical Analysis

Statistical analyses were performed using GraphPad Prism 5.0 (GraphPad Software, San Diego, USA). Survival curve comparison was determined using Log-rank Test. Comparison of the neurological impairment scores was performed using non-parametric test. The difference between two groups was compared using independent-samples T test. Multiple comparison procedures were carried out with one-way analysis of variance (ANOVA). The correlation between the percentage and absolute number of NK cells in different tissues was analyzed by linear correlation. The data are presented as the mean ± standard deviation (SD). A *P* value < 0.05 was considered statistically significant.

## Results

### NK Cells Infiltrate Into the CNS of Mice Infected With *A. cantonensis*

We constructed a mouse model of *A. cantonensis* infection by intragastric administration of third-stage larvae. To evaluate the pathological damage caused by *A. cantonensis* infection, the survival rate, body weight, neurological function, histological changes and cytokine levels in brain tissue were detected on 0, 10, 14, 18 and 22 dpi. As shown in **Figure S3**, infected mice showed decreased survival rate, reduced body weight, increased neurological dysfunction, aggravated tissue damage, and elevated levels of inflammatory cytokines (IL-1β, IL-6 and TNF-α) on 18 and 22 dpi, compared with that of 0 dpi.

**Figure 1:**
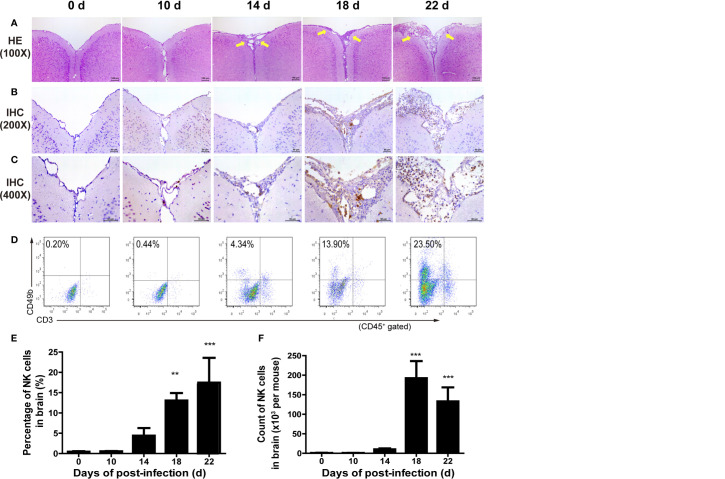
NK cells infiltrated into the CNS after *A. cantonensis* infection. **(A)** Representative histopathological sections of brain tissue in infected mice with H&E staining. The meninges of infected mice were damaged and inflammatory cells infiltrated under the meninges (yellow arrow) from 14 to 22 dpi. Images are shown at 100 × magnification (Scale bar, 100 µm). **(B, C)** Representative histopathological sections of brain tissue in infected mice with IHC staining. Brain sections were incubated with rabbit anti-mouse CD49b monoclonal antibody and stained with DAB. From 14 dpi to 22 dpi, more and more NK cells (stained brown) appeared under the meninges. Images are shown at 200 × and 400×magnification (Scale bar, 100 µm). **(D–F)** The percentage and absolute number of NK cells in brain mononuclear cells of infected mice. NK cells in brain mononuclear cells were detected by FCM. Data shown represent analysis from two independent experiments with three mice per group. Significance was determined by one-way ANOVA. ^**^ *P* < 0.01; ^***^ *P* < 0.001, compared with that of 0 dpi.

And then, we prepared the brain tissue sections with H&E staining. We observed that the meninges were damaged and more and more inflammatory cells infiltrated under the meninges from 14 to 22 dpi (**Figure 1A**). In order to detect the presence of NK cells in these infiltrating inflammatory cells, we performed IHC staining on brain tissue sections using anti-CD49b mAb as NK cell marker. **Figures 1B, C** showed that almost no NK cell was observed in the CNS on 0 dpi and 10 dpi. On 14 dpi, a small number of NK cells staining brown appeared under the meninges indicating that NK cells began to infiltrate into the CNS. On 18 dpi, more NK cells appeared in the CNS and the number of CNS-infiltrated NK cells peaked on 22 dpi. To monitor NK cells quantitatively, brain mononuclear cells were isolated and analyzed by FCM. As shown in **Figures 1D–F**, few NK cells were detected in the brain tissue at 0 dpi and 10 dpi. From 14 dpi to 22 dpi, the percentage and absolute number of CNS-infiltrated NK cells increased gradually with the extension of infection time. The highest percentage of CNS-infiltrated NK cells appeared on 22 dpi (22 dpi *vs* 0 dpi: 17.47 ± 6.11% *vs* 0.45 ± 0.12%, *P* < 0.001) and the maximum number of CNS-infiltrated NK cells were found on 18 dpi (18 dpi *vs* 0 dpi: 1.92 ± 0.43 × 10^5^ *vs* 696.90 ± 617.20 cells/mouse, *P <* 0.001).

**Figure 2:**
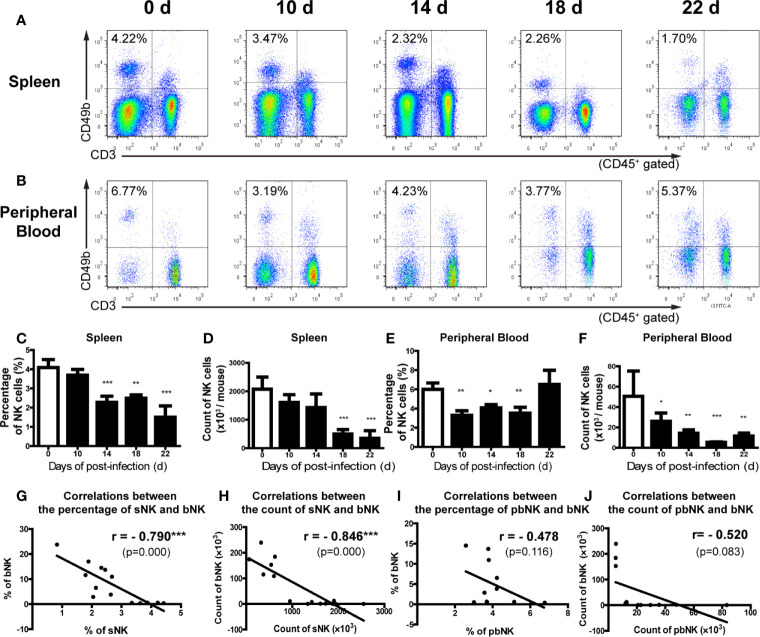
The percentage and absolute number of NK cells in spleen and peripheral blood decreased after *A. cantonensis* infection. **(A, C, D)** The percentage and absolute number of NK cells in splenic lymphocytes of infected mice. **(B, E, F)** The percentage and absolute number of NK cells peripheral blood mononuclear cells of infected mice. NK cells in the splenic lymphocytes and peripheral blood mononuclear cells were detected by FCM. **(G, H)** The correlation between the percentage and absolute number of NK cells in spleen and brain. **(I, J)** The correlation between the percentage and absolute number of NK cells in peripheral blood and brain. Data are expressed as the means ± SD. Data shown represent analysis from two independent experiments with three mice per group. Multiple comparisons of the percentage and count of NK cells at different time-points of infection were performed by one-way ANOVA. The correlation between the percentage and absolute number of NK cells in different tissues was analyzed by linear correlation. ^*^ *P* < 0.05; ^**^ *P* < 0.01; ^***^ *P* < 0.001, compared with that of 0 dpi. sNK, NK cells in spleen; bNK, NK cells in brain; pbNK, NK cells in peripheral blood.

**Figure 3:**
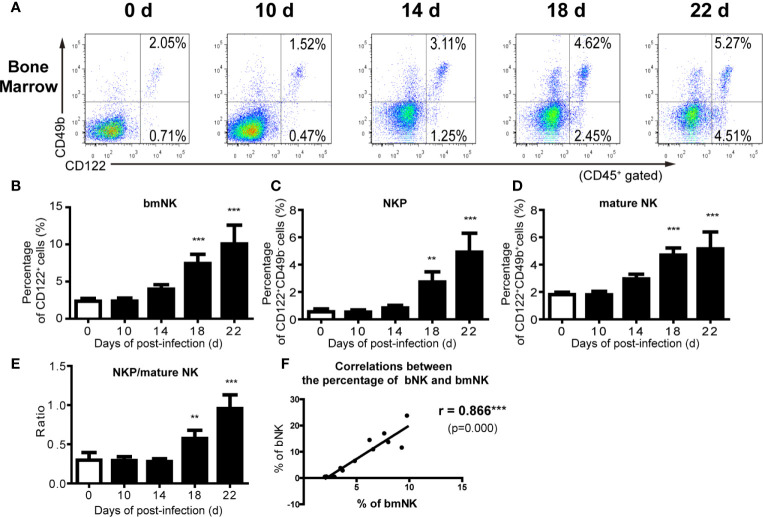
The percentage of NK cells in bone marrow increased after *A. cantonensis* infection. **(A–D)** The percentage of NK cells in bone marrow lymphocytes of *A. cantonensis*-infected mice. NK cells in bone marrow (CD122^+^) were divided into NK precursor cells (CD122^+^CD49b^-^) and mature NK cells (CD122^+^ CD49b^+^) detected by FCM. **(E)** The ratio of NK precursor cells to mature NK cells in bone marrow. **(F)** The correlation between the percentage of NK cells in bone marrow and brain. Data are expressed as the means ± SD. Data shown represent analysis from two independent experiments with three mice per group. Multiple comparisons of the percentage of NK cells at different time-points of infection were performed by one-way ANOVA. The ratio of different time points was analyzed by nonparametric test. The correlation between the percentage and number of NK cells in different tissues was analyzed by linear correlation. ^**^ *P* < 0.01; ^***^ *P* < 0.001, compared with that of 0 dpi. bmNK, NK cells in bone marrow; NKP, NK precursor cells; mature NK, mature NK cells.

We further analyzed the distribution of NK cells in the spleen, peripheral blood and bone marrow of *A. cantonensis*-infected mice. The results showed that the percentage and absolute number of NK cells in splenic lymphocytes decreased on 14, 18 and 22 dpi (**Figures 2A, C, D**). So did the absolute number of NK cells in PBMCs from 10 dpi to 22 dpi (**Figures 2B, E, F**). Furthermore, the percentage and absolute number of sNK (NK cells in spleen) were negatively correlated with those of bNK (NK cells in brain) (r =−;0.79, *P <* 0.01; r =−0.85, *P <* 0.01) (**Figures 2G, H**). The percentage and absolute number of pbNK (NK cells in PBMCs) and bNK were also negatively correlated, but there was no statistical significance (**Figures 2I, J**). However, the percentage of total NK cells (bmNK, CD122^+^), NK precursor cells (NKP, CD49b^-^CD122^+^) and mature NK cells (mature NK, CD49b^+^CD122^+^) in bone marrow all significantly increased on 18 and 22 dpi (**Figures 3A–D**). And the ratio of NKP to mature NK in bone marrow was up-regulated (**Figure 3E**). In addition, the percentage of total NK cells in bone marrow was positively correlated with that of bNK (r = 0.87, *P <* 0.001) (**Figure 3F**). Our results also showed that the percentage of T cells in splenic lymphocytes increased (22 dpi *vs* 0 dpi: 48.62 ± 6.65% *vs* 33.61 ± 2.23%, *P* < 0.01), while the absolute number of splenic T cells decreased with the extension of infection time (22 dpi *vs* 0 dpi: 9.77 ± 4.22 × 10^6^ *vs* 16.97 ± 2.74 × 10^6^ cells/mouse, *P* < 0.05) (**Figure S4**).

### CNS-Infiltrated NK Cells of *A. cantonensis*-Infected Mice Have Elevated Cytotoxicity and Secretory Ability

**Figure 4:**
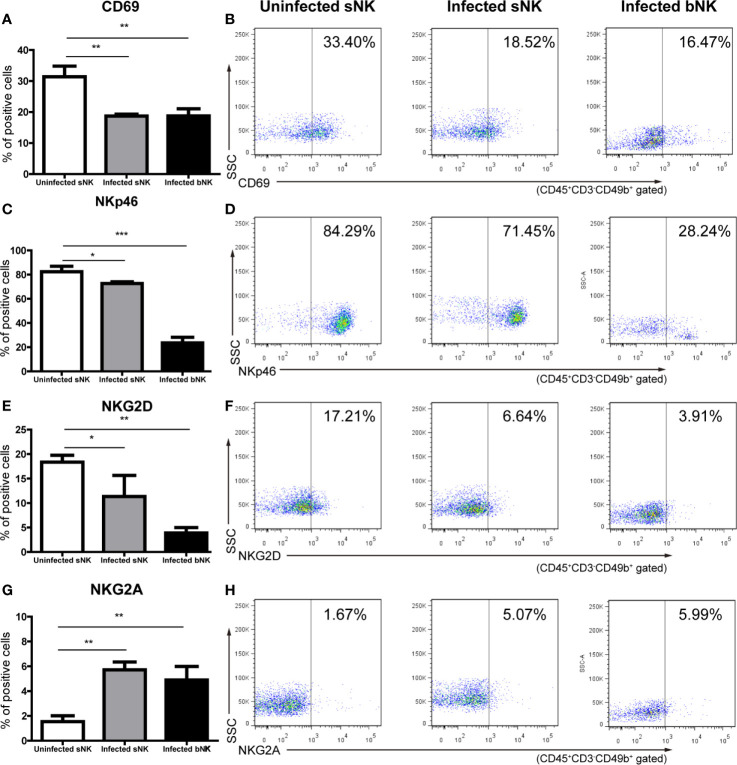
The phenotypes of NK cells changed after *A. cantonensis* infection. The expression of activation marker CD69 **(A, B)**, activated receptor NKp46 **(C, D)** and NKG2D **(E, F)**, and inhibitory receptor NKG2A **(G, H)** on the surface of sNK and bNK were detected by FCM. Gating strategy for the mouse CD45^+^CD3^-^CD49b^+^ NK cell population was shown in **Figure S2**. Representative dot plots stained with isotype controls was shown in **Figure S5**. Data are expressed as the means ± SD. Data shown represent analysis from two independent experiments with three mice per group. Multiple comparisons of phenotypes between uninfected sNK, infected sNK and infected bNK were performed by ANOVA. ^*^ *P* < 0.05; ^**^ *P* < 0.01; ^***^ *P* < 0.001. sNK, NK cells in spleen; bNK, NK cells in brain.

We further detected the phenotypic and functional changes of NK cells after *A. cantonensis* infection. We measured the expression of activation marker CD69, activated receptor NKp46 and NKG2D, and inhibitory receptor NKG2A on NK cells by FCM. **Figure 4** revealed that infected bNK and infected sNK expressed lower levels of CD69, NKp46 and NKG2D, but higher levels of NKG2A, compared with uninfected sNK. Although the phenotypic changes of infected bNK were greater than those of infected sNK, there was no statistical difference between them.

**Figure 5:**
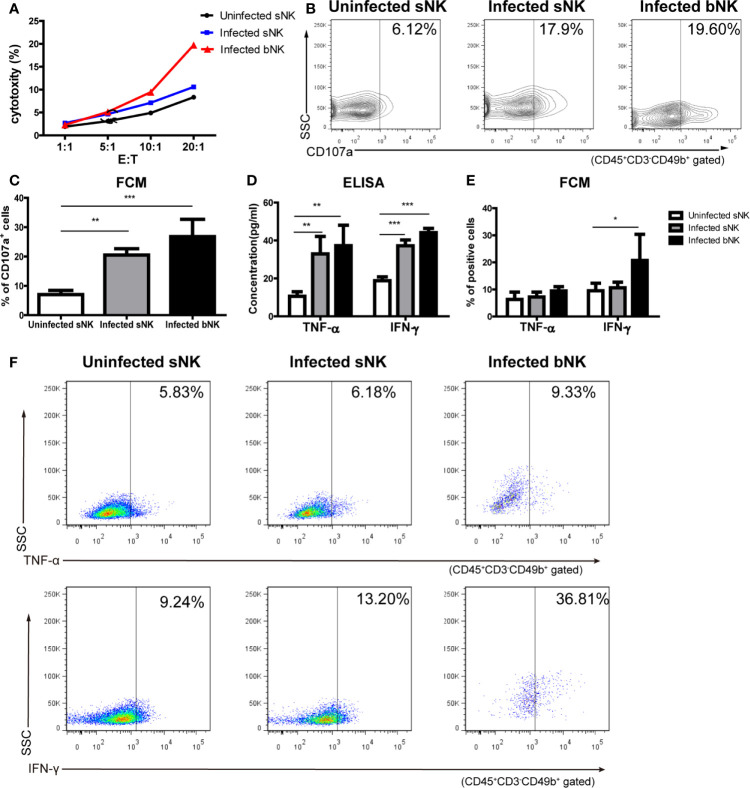
The cytotoxicity and secretory ability of NK cells elevated after *A. cantonensis* infection. **(A)** NK cell-mediated cytotoxicity against YAC-1 cells. Purified sNK and bNK (as effector cells) were incubated with YAC-1 cells (as target cells) at various effector cell/target cell ratios (E:T = 1:1, 5:1, 10:1, 20:1). NK cell-mediated cytotoxicity was detected by LDH release assay. **(B, C)** The expression of CD107a on NK cells. Splenic lymphocytes and brain mononuclear cells were isolated and detected by FCM. Gating strategy for the mouse CD45^+^CD3^-^CD49b^+^ NK cell population was shown in **Figure S2**. **(D)** The concentrations of TNF-α and IFN-γ in the culture supernatants of NK cells. Purified sNK and bNK were stimulated with IL-12 and the concentrations of TNF-α and IFN-γ in the culture supernatants were detected using ELISA. **(E, F)** The expression of intracellular cytokine TNF-α and IFN-γ in NK cells. Splenic lymphocytes and brain mononuclear cells were isolated and stimulated with Leukocyte Activation Cocktail. The percentage of TNF-α^+^ cells and IFN-γ^+^ cells in CD45^+^CD3^-^CD49b^+^ NK cells was detected by FCM. Data are expressed as the means ± SD. Data shown represent analysis from two independent experiments with four mice per group. Multiple comparisons were performed by one-way ANOVA. ^*^ *P* < 0.05; ^**^ *P* < 0.01; ^***^ *P* < 0.001. sNK, NK cells in spleen; bNK, NK cells in brain.

Then we detected NK cell-mediated cytotoxicity against YAC-1 cells by LDH release assay. As shown in **Figure 5A**, infected bNK and infected sNK had enhanced cytotoxicity, compared with uninfected sNK (infected bNK *vs* infected sNK *vs* uninfected sNK: 19.37% *vs* 10.59% *vs* 8.35%, as Effect cells: Target cells = 20:1). Furthermore, the expression of CD107a, a surface marker of NK cell degranulation, on infected bNK and infected sNK was up-regulated compared with that of uninfected sNK (**Figures 5B, C**).

The ability of NK cells to secrete cytokines was determined by two methods: ELISA (secretory levels) and FCM (intracellular levels). Firstly, we detected the concentrations of TNF-α and IFN-γ in the culture supernatant of NK cells after IL-12 stimulation using ELISA. The levels of TNF-α and IFN- γ secreted by infected bNK and infected sNK were significantly higher than those of uninfected sNK (**Figure 5D**). Subsequently, purified NK cells were stimulated and measured for intracellular cytokine by FCM. The percentage of IFN-γ^+^ cells in infected bNK was higher than that of uninfected sNK, while the percentage of TNF-α^+^ or IFN-γ^+^ cells in infected sNK did not change (**Figures 5E, F**).

### NK Cells Aggravate Brain Injury of Mice Caused by *A. cantonensis* Infection

**Figure 6:**
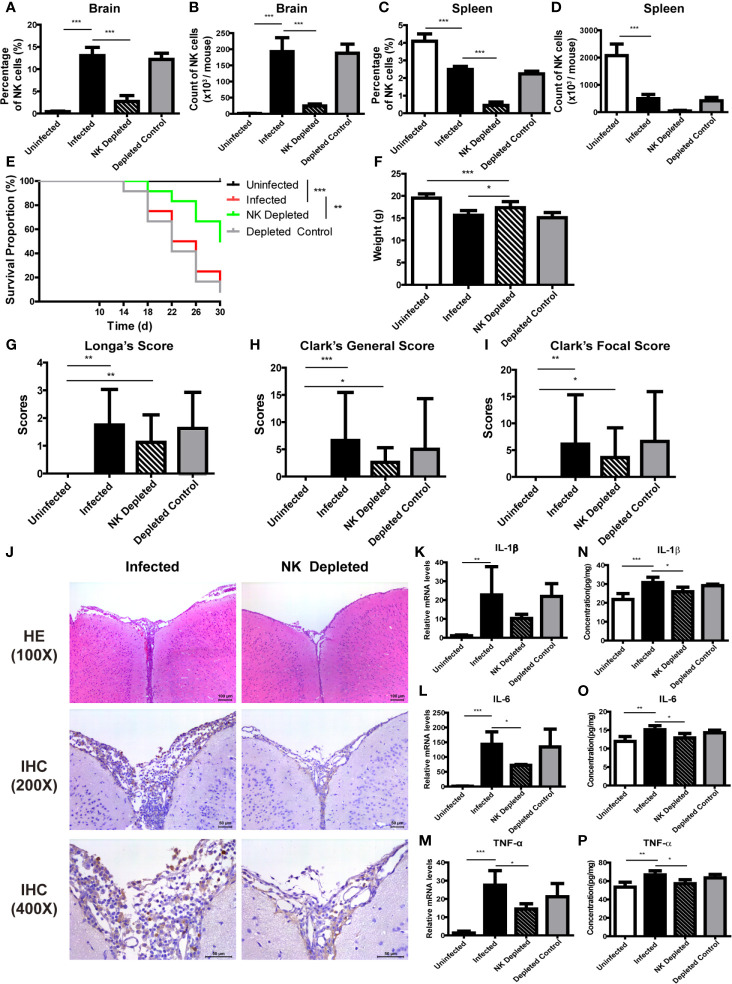
Depletion of NK cells alleviated brain injury in *A*. *cantonensis*-infected mice. Depletion of NK cells in infected mice was induced by tail vein injection of anti-asialo GM1 rabbit serum. The percentage and absolute number of NK cells in brain **(A, B)** and spleen **(C, D)** were detected by FCM to evaluate NK cell depleting efficiency. After NK cell depletion, the survival rate of infected mice increased **(E)**, body weight elevated **(F)**, while neurological impairment scores did not have significant changes **(G–I)**. Representative histopathological sections of brain tissue with H&E and IHC staining showed alleviated inflammation and fewer infiltrating NK cells in the brain of NK-depleted mice **(J)**. Images are shown at 100 ×, 200 × and 400 × magnification (Scale bar, 50-100 µm). The gene **(K–M)** and protein levels **(N–P)** of inflammatory cytokines IL-1β, IL-6 and TNF-α in brain tissue reduced after NK cell depletion measured by qRT-PCR and ELISA. Data are expressed as the means ± SD. Data shown represent analysis from two independent experiments with three to twelve mice per group. Survival curve comparison was determined by Log-rank Test. Comparison of the neurological impairment scores was compared by non-parametric test. Multiple comparisons of the percentage and absolute number of NK cells, body weight and expression of cytokines were performed using one-way ANOVA. ^*^ *P* < 0.05; ^**^ *P* < 0.01; ^***^ *P* < 0.001. Uninfected, normal mice; Infected, mice infected with *A*. *cantonensis*; NK Depleted, infected mice depleted NK cells by injection of anti-asialo GM1 rabbit serum; Depleted Control, infected mice given normal rabbit serum as control.

We designed NK cell depletion and adoptive transfer experiments to elucidate the role of NK cells in brain injury induced by *A. cantonensis* infection. Firstly, NK cells were depleted by tail vein injection of anti-asialo GM1 serum to infected mice. The percentage and absolute number of NK cells in brain and spleen of infected mice significantly decreased after NK cell depletion (**Figures 6A–D, J**). Survival rate of the NK-depleted mice increased (*P <* 0.01), body weight elevated (NK-depleted *vs* infected: 17.35 ± 1.38 g *vs* 15.61 ± 1.10 g, *P <* 0.05), neurological impairment score decreased slightly, brain tissue inflammation alleviated, and the expression levels of inflammatory cytokines in brain tissue reduced (NK-depleted *vs* infected: IL-1β 25.88 ± 2.38 pg/mg *vs* 30.75 ± 2.83 pg/mg, IL-6 12.88 ± 1.23 pg/mg *vs* 15.08 ± 1.13 pg/mg, TNF-α 57.17 ± 4.20 pg/mg *vs* 64.68 ± 4.34 pg/mg, *P <* 0.05), compared with the infected mice (**Figures 6E–P**).

**Figure 7:**
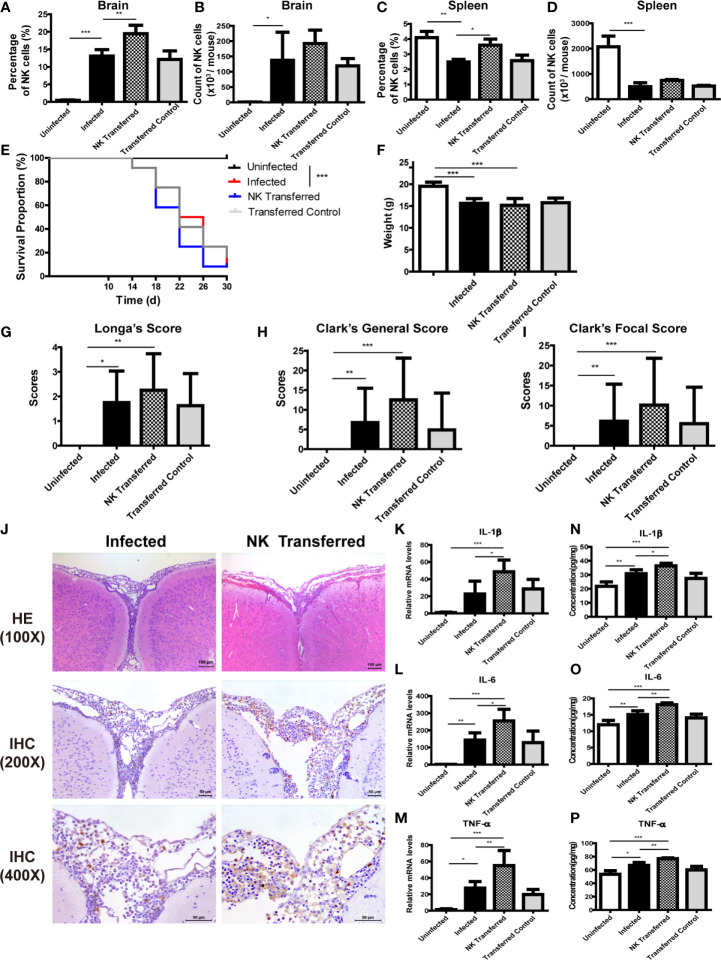
Adoptive transfer of NK cells exacerbated brain damage in *A. cantonensis*-infected mice. Purified splenic NK cells from normal mice were transferred to infected mice by tail vein injection. The percentage and absolute number of NK cells in brain **(A, B)** and spleen **(C, D)** were detected by FCM to evaluate NK cell adoptive transferring efficiency. After adoptive transferring NK cells, the survival rate of infected mice **(E)**, body weight **(F)** and neurological impairment score **(G–I)** did not change significantly. Representative histopathological sections of brain tissue with H&E and IHC staining showed aggravated inflammation and more infiltrating NK cells in the brain of NK- transferred mice **(J)**. Images are shown at 100 ×, 200 × and 400 × magnification (Scale bar, 50-100 µm). The gene **(K–M)** and protein levels **(N–P)** of inflammatory cytokines IL-1β, IL-6 and TNF-α in brain tissue elevated after NK cell adoptive transferring measured by qRT-PCR and ELISA. Data are expressed as the means ± SD. Data shown represent analysis from two independent experiments with three to twelve mice per group. Survival curve comparison was determined by Log-rank Test. Comparison of the neurological impairment scores was compared by non-parametric test. Multiple comparisons of the percentage and absolute number of NK cells, body weight and expression of cytokines were performed using one-way ANOVA. ^*^ *P* < 0.05; ^**^ *P* < 0.01; ^***^ *P* < 0.001. Uninfected, normal mice; Infected, mice infected with *A*. *cantonensis*; NK Transferred, infected mice transferred NK cells by tail vein injection; Transferred Control, infected mice injected with PBS as control.

And then, purified splenic NK cells were transferred to infected mice by tail vein injection. The percentage of NK cells in brain and spleen of infected mice increased significantly after adoptive transferring NK cells (**Figures 7A–D, J**). Compared with the infected mice, survival rate, body weight and neurological impairment score of the NK-transferred mice did not change significantly, but brain tissue inflammation aggravated, and the expression levels of inflammatory cytokines in brain tissue elevated (NK-transferred *vs* infected: IL-1β 36.36 ± 1.89 pg/mg *vs* 30.75 ± 2.83 pg/mg, *P <* 0.05; IL-6 18.08 ± 0.55 pg/mg *vs* 15.08 ± 1.13 pg/mg, *P <* 0.01; TNF-α 76.84 ± 1.21 pg/mg *vs* 64.68 ± 4.34 pg/mg, *P <* 0.01) (**Figures 7E–P**).

### CX_3_CL1 Recruits NK Cells Into the CNS of Mice After *A. cantonensis* Infection

**Figure 8:**
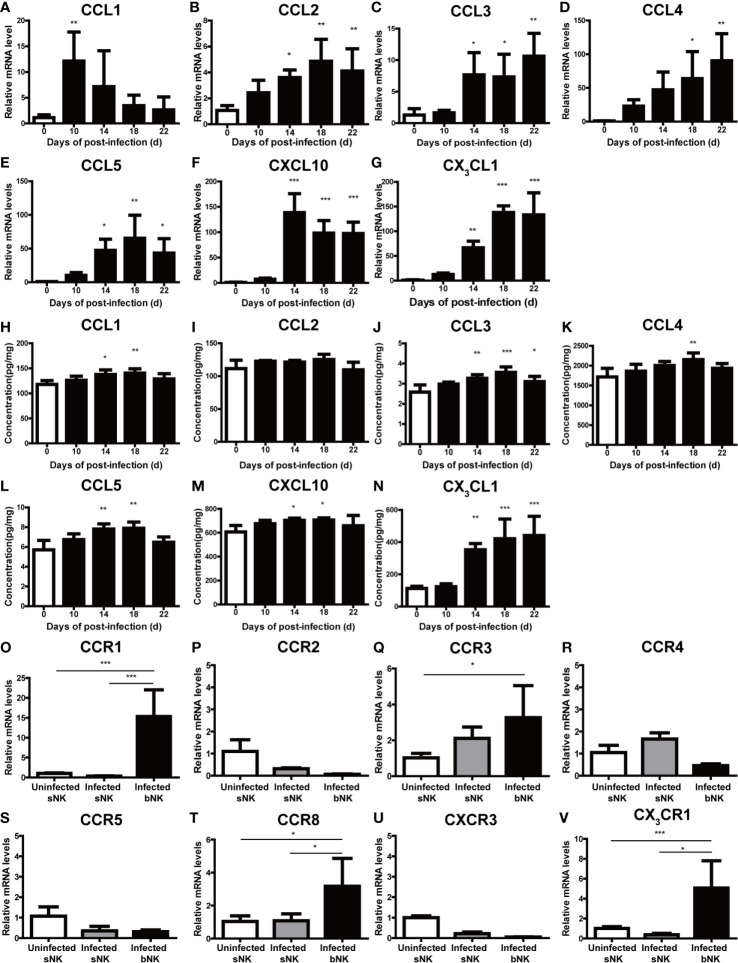
The expression of CX_3_CL1 in the brain tissue and CX_3_CR1 on the CNS-infiltrated NK cells were elevated after *A. cantonensis* infection. The gene **(A–G)** and protein levels **(H–N)** of various chemokines (CCL1, CCL2, CCL3, CCL4, CCL5, CXCL10 and CX_3_CL1) in brain tissue were measured by qRT-PCR and ELISA. **(O–V)** The gene expression levels of chemokine receptors (CCR1, CCR2, CCR3, CCR4, CCR5, CCR8, CXCR3 and CX_3_CR1) on NK cells were detected by qRT-PCR. Multiple comparisons were performed by ANOVA. ^*^ *P* < 0.05; ^**^ *P* < 0.01; ^***^ *P* < 0.001. sNK, NK cells in spleen; bNK, NK cells in brain.

To elucidate the key factors that recruited NK cells into the CNS of *A. cantonensis*-infected mice, we detected the expression of various chemokines in brain tissue. As shown in **Figures 8A–N**, the expression levels of CCL3, CCL5, CXCL10 and CX_3_CL1 in the brain tissue of infected mice was elevated with the extension of infection time. Among them, CX_3_CL1 showed the most significant change. The gene and protein expression levels of CX_3_CL1 were both significantly increased on 18 dpi compared with those of 0 dpi (18 dpi *vs* 0 dpi: mRNA 138.00 ± 13.3 *vs* 1.11 ± 0.59, protein 441.40 ± 118.70 *vs* 112.60 ± 13.38 pg/mg, *P* < 0.001). And then, we analyzed the expression of chemokine receptors on NK cells. The gene expression levels of CCR1(the receptor of CCL5), CCR8 (the receptor of CCL1) and CX_3_CR1 (the receptor of CX_3_CL1) on infected bNK was significantly elevated, compared with that of uninfected sNK or infected sNK (**Figures 8O–V**). Taken together, the expression of CX_3_CL1 in the brain tissue and its receptor CX_3_CR1 on the CNS-infiltrated NK cells were both upregulated after *A. cantonensis* infection.

**Figure 9:**
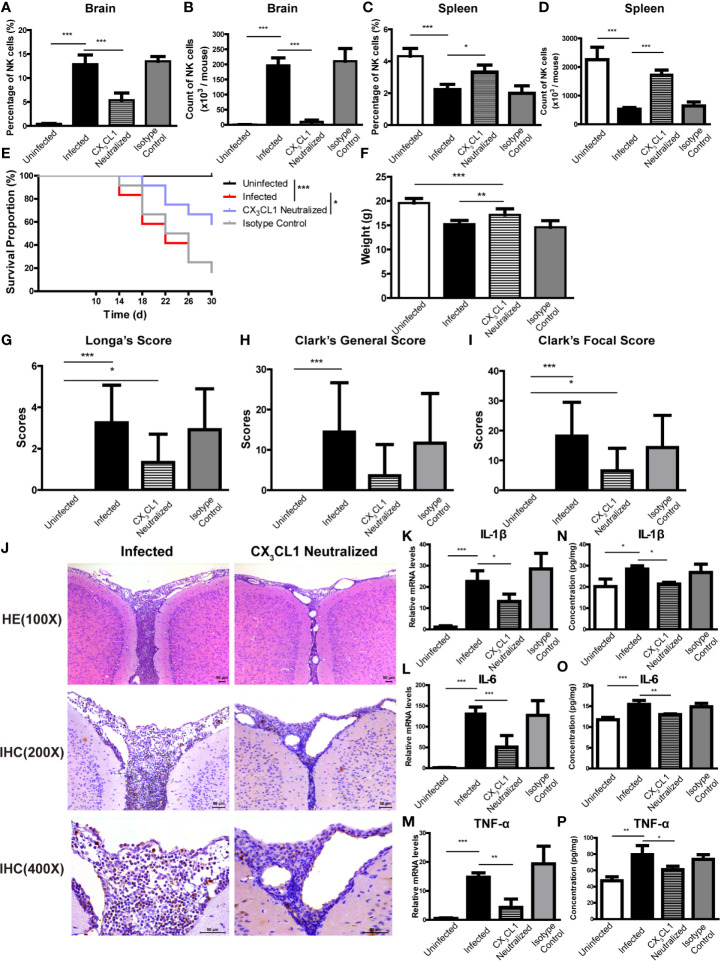
CX_3_CL1 neutralization reduced the infiltration of NK cells into CNS and relieved brain damage caused by *A*. *cantonensis* infection. Infected mice were given daily intraperitoneal injections of anti-CX_3_CL1 IgG or isotype control IgG from 10 dpi to 17 dpi and euthanatized at 18 dpi. After CX_3_CL1 neutralization, the percentage and absolute number of NK cells in brain **(A, B)** of infected mice significantly decreased, while the percentage and absolute number of NK cells in spleen **(C, D)** significantly increased detected by FCM. The CX_3_CL1 neutralized mice showed elevated survival rate **(E)**, increased body weight **(F)**, slightly decreased neurological impairment score **(G–I)**, compared with the infected mice. Representative histopathological sections of brain tissue with H&E and IHC staining showed alleviated inflammation and fewer infiltrating NK cells in the brain of CX_3_CL1 neutralized mice **(J)**. Images are shown at 100 ×, 200 × and 400 × magnification (Scale bar, 50-100 µm). The gene **(K–M)** and protein levels **(N–P)** of inflammatory cytokines IL-1β, IL-6 and TNF-α in brain tissue reduced after CX_3_CL1 neutralization measured by qRT-PCR and ELISA. Data are expressed as the means ± SD. Data shown represent analysis from two independent experiments with three to twelve mice per group. Survival curve comparison was determined by Log-rank Test. Comparison of the neurological impairment scores was compared by non-parametric test. Multiple comparisons of the percentage and absolute number of NK cells, body weight and expression of cytokines were performed using one-way ANOVA. ^*^ *P* < 0.05; ^**^ *P* < 0.01; ^***^ *P* < 0.001. Uninfected, normal mice; Infected, mice infected with *A. cantonensis*; CX_3_CL1 Neutralized, infected mice inject with anti-CX_3_CL1 IgG; Isotype Control, infected mice given isotype control IgG.

To determine if CX_3_CL1 activity is important for the recruitment of NK cells into the CNS, *A. cantonensis*-infected mice were given daily intraperitoneal injections of anti-CX_3_CL1 IgG (neutralizing antibody) or an isotype control antibody starting at 10 dpi (before the increase of CX_3_CL1 in brain tissue of infected mice shown in **Figures 8G, N**) and euthanatized at 18 dpi. After CX_3_CL1 neutralization, the percentage and absolute number of NK cells in brain of infected mice significantly decreased (CX_3_CL1 neutralized *vs* infected: 5.38 ± 1.49% *vs* 12.90 ± 1.91%, *P* < 0.001; 9.88 ± 5.78 × 10^3^ *vs* 1.96 ± 0.24 × 10^5^ cells/mouse, *P* < 0.001), while the percentage and absolute number of NK cells in spleen significantly increased (CX3CL1 neutralized *vs* infected: 3.32 ± 0.43% *vs* 2.22 ± 0.32%, *P* < 0.05; 1.73 ± 0.16 × 10^6^ *vs* 0.52 ± 0.05 × 10^6^ cells/mouse, *P* < 0.001) (**Figures 9A–D, J**). The CX_3_CL1 neutralized mice showed elevated survival rate (*P <* 0.05), increased body weight (CX_3_CL1 neutralized *vs* infected: 17.13 ± 1.26 g *vs* 15.22 ± 0.75 g, *P <* 0.01), slightly decreased neurological impairment score, alleviated brain tissue inflammation, and reduced expression levels of inflammatory cytokines (CX_3_CL1 neutralized *vs* infected: IL-1β 21.36 ± 0.77 pg/mg *vs* 28.37 ± 1.58 pg/mg, *P <* 0.05; IL-6 12.97 ± 0.19 pg/mg *vs* 15.45 ± 0.95 pg/mg, *P <* 0.01; TNF-α 60.76 ± 4.28 pg/mg *vs* 79.41 ± 11.15 pg/mg, *P <* 0.05), compared to the infected mice (**Figures 9E–P**).

## Discussion

Angiostrongylosis, a food-borne parasitic disease, is caused by the larvae of *A. cantonensis* in the host’s central nervous system (Martins et al., 2015). NK cells are important innate immune effector cells. They can be swiftly mobilized by danger signals and are among the earliest arrivals at target organs against pathogen infection (Cruzmunoz and Veillette, 2010). However, the role of NK cells in the CNS damage caused by *A. cantonensis* infection remains elusive. Our previous work has reported that NK cells in the spleen and peripheral blood showed quantitative reduction and functional changes in an *A. cantonensis*-infected mice model (Chen et al., 2014). In the current study, we focused on the NK cells in the CNS. We found a large number of NK cells infiltrated into the CNS of mice after *A. cantonensis* infection and these CNS-infiltrated NK cells had elevated cytotoxicity and secretory ability. Moreover, we demonstrated that the increased expression of CX_3_CL1 in the brain tissue recruited NK cells into the CNS and aggravated brain injury of mice caused by *A. cantonensis* infection.

Mice and humans are both non-permissive hosts of *A. cantonensis*, and the pathogenic process is comparatively similar (Ouyang et al., 2012). Consistent with other reports (Guo et al., 2008; Wang et al., 2015; Chen et al., 2016), we successfully constructed a mouse model infected with *A. cantonensis* and observed serious neurological damage from 18 dpi to 22 dpi. What is noteworthy in this study is that we confirmed that NK cells infiltrated into the CNS after *A. cantonensis* infection. The results of IHC and FCM showed that NK cells began to appear in the brain tissues at 14 dpi. The percentage and absolute number of NK cells increased gradually with the extension of infection time until 22 dpi. The CNS, including the brain and spinal cord, is considered as an immune privileged organ because of the low permeability of the BBB. However, it is conceivable that peripherally activated lymphocytes, including NK cells, might also be able to penetrate the BBB and infiltrate into the CNS under several pathological conditions. In human ischemic brain tissue and a permanent middle cerebral artery occlusion (pMCAO) mouse model, infiltration of NK cells into the ischemic infarct region are observed (Gan et al., 2014; Zhang et al., 2014; Li et al., 2020). NK cells are activated in the periphery and then migrated into the CNS of EAE mice (Hao et al., 2010). NK cells can be detected in mouse CNS tissues during a variety of infections, including Semliki Forest virus (SFV) (Alsharifi et al., 2006), murine coronavirus (Hayashi et al., 2009) and *L. monocytogenes* (Trifilo et al., 2004). NK cells are also recruited to the CNS in glioma-bearing mice and constitute approximately 50% of all leukocytes in the CNS (Alizadeh et al., 2010). We further examined the distribution of NK cells in the spleen, peripheral blood and bone marrow of *A. cantonensis*-infected mice. The results showed that the percentage and absolute number of NK cells in spleen and in peripheral blood both decreased, consistent with our previous study (Chen et al., 2014), while the percentage of NK cells in bone marrow increased after *A. cantonensis* infection. In addition, the percentage and absolute number of splenic NK cells were negatively correlated with those of brain NK cells, while the percentage of NK cells in bone marrow was positively correlated with brain NK cells. It was suggested that the CNS-infiltrated NK cells probably were related with the increased hematopoiesis of bone marrow and migration of peripheral NK cells after *A. cantonensis* infection.

To identify the characteristics of the CNS-infiltrated NK cells, we detected the phenotype and function of NK cells after *A. cantonensis* infection. The results showed that the expression of activation molecule CD69, activating receptor NKp46 and NKG2D on the CNS-infiltrated NK cells of infected mice was decreased compared with the splenic NK cells of uninfected mice, while the expression of inhibitory receptor NKG2A increased. Consistent phenotypic changes were observed in the splenic NK cells of infected mice. CD69, as an early activation marker on NK cells, is also a novel immune regulator, which can inhibit the cytotoxicity of NK cells by inducing the production of TGF-β. It was demonstrated that the administration of anti-CD69 mAbs can activate resting NK cells, resulting in a substantial increase in both NK-cell cytolytic activity and IFN-γ production (Esplugues et al., 2005). NK cells express an array of inhibitory and activating receptors recognizing self-ligands or microbial molecules on infected and tumor cells. Coordinated acquisition of these inhibitory and activating signals regulates the effector functions of NK cells (Vivier et al., 2011). Some studies have reported the phenotypic changes in the CNS-infiltrated NK cells under pathological conditions. In the EAE mice, the CNS-infiltrated NK cells upregulate the inhibitory receptor NKG2A and kill reactive CD4^+^T cells (Hao et al., 2010). In a mouse model of cerebral ischemia, NK cells in the ischemic hemisphere have increased expression of NKG2D, an activation receptor, while similar expression of NKG2A, an inhibitory receptor. Of note, expression of the MHC-Ib molecule Qa1, the ligand for NKG2A, decreased significantly on ischemic neurons (Gan et al., 2014). NK cell-mediated neuronal damage is associated with the loss of self-identity for ischemic neuron-modulated NK cell tolerance and the activation of NK cells. The down-regulation of CD69 and activating receptors and up-regulation of inhibitory receptors on NK cells following *A. cantonensis* infection might imply the changes of their effector functions.

NK cells have a variety of biological functions, with the most important role being cytotoxicity (Abel et al., 2018). In our study, we found that the CNS-infiltrated NK cells of infected mice had an enhanced cytotoxicity against YAC-1 cells with higher expression of CD107a. CD107a, also known as Lysosome associated membrane protein-1 (LAMP-1), is a marker for degranulation of NK cells and its expression correlates with NK cell-mediated lysis of target cells (Alter et al., 2004; Aktas et al., 2009). NK cells can also produce a variety of cytokines in response to activation signaling to regulate immune response. Our results showed that compare with the splenic NK cells of uninfected mice, the CNS-infiltrated NK cells of infected mice produced higher levels of TNF-α and IFN-γ, which are both proinflammatory cytokines and involved in mediating anti-pathogen immune responses (Schoenborn and Wilson, 2007; Zou et al., 2010). Taken together, the CNS-infiltrated NK cells in *A. cantonensis*-infected mice showed stronger activity with enhanced cytotoxicity and elevated production of TNF-α and IFN-γ. There are no NK cells in the steady-state CNS, but NK cells might migrate into the CNS under certain pathological conditions. After homing to the inflamed CNS, NK cells become receptive to an array of cellular components that they have not encountered in the periphery. These include astrocytes, microglia, neurons and other cells, which release numerous soluble factors with diversified and perhaps coordinated effects on NK cells (Shi et al., 2011). The fate and function of NK cells are determined by focal environmental factors (Li et al., 2020). The exact cellular and molecular interactions that shape the phenotype and function of NK cells in the CNS still need to be determined.

We then investigated the role of NK cells in the brain damage caused by *A. cantonensis* infection using NK cell depletion and adoptive transfer experiments. We found that after NK depletion, the survival rate and body weight increased, nerve injury and brain inflammation decreased in *A. cantonensis*-infected mice. On the contrary, the inflammation in brain aggravated after adoptive transfer of NK cells. These results indicated that the CNS-infiltrated NK cells might exacerbated the brain injury after *A. cantonensis* infection. Many studies have reported that NK cells can rapidly accumulated into the CNS under pathological conditions (Alsharifi et al., 2006; Hayashi et al., 2009; Hao et al., 2010; Gan et al., 2014; Li et al., 2020). However, the role of NK cells in brain injury diseases remains is complex and sometimes paradoxical. Li et al. (2020) identified, NK cells infiltrate into the CNS during early stages of intracerebral hemorrhage (ICH), express up-regulated CD69 and perforin and exacerbate brain edema *via* cytotoxicity toward cerebral endothelial cells and recruitment of neutrophils. Gan et al. (2014) reported NK cells with the increased expression of NKG2D and IFN-γmediate exacerbation of brain infarction after ischemia *via* the disruption of NK cell tolerance, augmenting local inflammation and neuronal hyperactivity. Alsharifi et al. (2006) found that NK cells exert both disease-exacerbating and protective effects in the CNS of SFV-infected in mice. The cytolytic activity of NK cells is detrimental, while IFN-γ production is beneficial for recovery from SFV infection. However, Hao et al. (2010) demonstrated that the CNS-resident NK cells have a protective role in the brain of EAE mice, as they inhibit the activation of autoimmune T cells through the killing of activated microglia. Jiang et al. (2017) disclosed that acetylcholine-producing NK cells attenuate CNS inflammation of EAE model *via* modulation of infiltrating monocytes/macrophages. NK cells play different roles in brain injury, which may be related to the initial factors of the primary disease, the time of immune response, and the overall inflammatory process (Gan et al., 2014).

NK cells originate from bone marrow, are mainly distributed in peripheral blood and spleen, and some lymphatic tissues (Abel et al., 2018). However, the distribution of NK cells is not static because these cells can recirculate between organs. NK cells can respond to a large array of chemokines and be recruited to distinct sites in several pathological circumstances (Shi et al., 2011). The detailed trafficking patterns of NK cells are not very well characterized. Nevertheless, it appears that chemokines produced by cells that are unique to specific organs may have a role in orchestrating NK cell migration to each organ (Shi et al., 2011). It was reported that NK cells might be recruited to the CNS by chemokines such as CX_3_CL1 produced by neurons (Gan et al., 2014; Hertwig et al., 2016) and CCL2 and CXCL10 produced by microglia, astrocytes or infiltrating inflammatory cells (Hao et al., 2011; Zhang et al., 2014). We examined the expression levels of various chemokines (CCL1, CCL2, CCL3, CCL4, CCL5, CXCL10 and CX_3_CL1) in brain tissue and their corresponding receptors on NK cells of *A. cantonensis*-infected mice. The upregulation of CX_3_CL1 in the brain tissue and its receptor CX_3_CR1 on the CNS-infiltrated NK cells of infected mice indicated that CX_3_CL1 might be involved in the recruitment of NK cells into the CNS after *A. cantonensis* infection.

CX_3_CL1, also known as fractalkine (in human) and neurotactin (in mouse), is a large cytokine protein of 373 amino acids and is the only member of the CX_3_C chemokine family (Poniatowski et al., 2017). CX_3_CL1 have two forms: membrane-bound and soluble type. Soluble CX_3_CL1 potently chemoattracts T cells, monocytes, NK cells and other lymphocytes, while the membrane-bound CX_3_CL1 promotes strong adhesion of leukocytes to activated endothelial cells, where it is primarily expressed (Lee et al., 2018). CX_3_CL1 discloses its biological properties through interaction with one dedicated chemokine receptor CX_3_CR1 (Sheridan and Murphy, 2013). Some studies have reported the CX_3_CL1/CX_3_CR1 signal mediates NK cell migration from the periphery to the CNS. Huang et al. (2006) firstly reported that chemokine CX_3_CL1 selectively recruits NK cells to the CNS and modify experimental autoimmune encephalomyelitis. Hertwig et al. (2016) showed that mature NK cells are mobilized from the periphery and accumulate in the inflamed CNS of EAE mice in a CX_3_CR1-dependent way and contributes to control autoimmune neuroinflammation. Gan Y (Gan et al., 2014) demonstrated that ischemic neurons are the major source of CX_3_CL1 in the brain and the neuron-derived CX_3_CL1 recruits CX_3_CR1-expressing NK cells to the infarct sites, determining the sizes of brain lesions in a mouse model of cerebral ischemia. In our study, we found that the neutralization of CX_3_CL1 reduced the percentage and absolute number of NK cells in the CNS of *A. cantonensis*-infected mice, whereas increased the percentage and absolute number of the splenic NK cells. NK cells are a special heterogeneous population. The NK cells in different tissues and organs have divergent phenotypic and functional features, and are recruited by different chemokines (Shi et al., 2011). CX_3_CL1 neutralization only reduced the migration of brain NK cells in *A. cantonensis*-infected mice but did not inhibit the recruitment of splenic NK cells. After the neutralization of CX_3_CL1, infected mice had elevated survival rate, increased body weight, slightly decreased neurological impairment score, alleviated brain tissue inflammation, and reduced expression levels of inflammatory cytokines. These results suggest that CX_3_CL1 plays an important role in the recruitment of NK cells into the CNS and the progression of brain injury caused by *A. cantonensis* infection. We hypothesize that *A. cantonensis* larvae enter the brain tissue and induce mechanical damage and inflammatory response. Worm antigen and excretory-secretory antigens stimulate neurons to produce CX_3_CL1, which recruits CX_3_CR1^+^ NK cells in peripheral blood across the damaged BBB into the CNS. The inflammatory environment in the brain shapes NK cell new features with enhanced cytotoxicity and increased cytokine secretion ability, which causing immunopathologic damage while clearing pathogens, and eventually exacerbate brain injury.

Currently, the treatment for angiostrongyliasis includes supportive treatment and corticosteroid therapy (Graeff-Teixeira et al., 2018). The use of anthelmintic drugs, such as albendazole and mebendazole, to kill worms remains controversial. The dead worm lysis in the CNS might cause severe inflammatory response and further damage (Lv et al., 2017). Meanwhile, Patients under the treatment of high-dose corticosteroids would experience immune suppression (Cowie, 2017). Therefore, it is urgent to develop new therapeutic interventions for angiostrongyliasis. Recently, the selective blockage of disease-relevant chemokines/chemokines receptors has become a new treatment strategy and has been proved to be effective in various diseases (Miao et al., 2020). Three chemokine antagonists have been approved: Maraviroc (a CCR5 antagonist) for anti-HIV treatment (Xu et al., 2014), Plerixafor (a CXCR4 antagonist) for the treatment of multiple myeloma or non-Hodgkin’s lymphoma (Uy et al., 2008), and Mogamulizumab (a CCR4 antagonist) for the treatment of mycosis fungoides or Sézary syndrome (Sato et al., 2018). Moreover, clinical trials are ongoing to evaluate many potent candidates. For example, E6011 is a novel humanized anti-CX_3_CL1 monoclonal antibody being developed as a therapeutic target for Crohn’s disease, RA, and primary biliary cholangitis (Tabuchi et al., 2019). We speculated that selective blockage of NK cell infiltration into the CNS may help to alleviate the brain injury caused by *A. cantonensis* infection or other pathogens, which need to be further investigated in our future work.

In conclusion, our study demonstrates that NK cells infiltrate into the CNS of *A. cantonensis*-infected mice. These CNS-infiltrated NK cells display enhanced cytotoxicity and secretory ability. The up-regulated CX_3_CL1 in the brain tissue recruits NK cells into the CNS and aggravates brain damage caused by *A. cantonensis* infection. Our findings not only enrich the understanding of the pathogenesis of angiostrongylosis but also provide a clue to novel potential therapeutic strategies against CNS disease.

## Data Availability Statement

The original contributions presented in the study are included in the article/**Supplementary Material**. Further inquiries can be directed to the corresponding authors.

## Ethics Statement

The animal study was reviewed and approved by the Institutional Animal Care and Use Committee of Nanjing Medical University.

## Author Contributions

AC conceived the project and designed the experiments. YW designed the experiments, supervised the project, and was involved in all aspects of the submission. RZ performed most of the experiments, analyzed data, and wrote the manuscript. TM was responsible for the infection and feeding of animals, performed cell isolation and detection by FCM. MQ and CSZ performed detection of cytokine by qRT-PCR and ELISA. WW and CCZ participated in neurological impairment evaluation and NK cell cytotoxicity assays. XL and YC performed histopathological examination and data analysis. All authors contributed to the article and approved the submitted version.

## Funding

This work was supported by the National Natural Science Foundation of China (No. 81501371), the Post doctorate Foundation of China (2019M651963), the Post doctorate Foundation of Jiangsu Province (2018Z093), Jiangsu Provincial Medical Youth Talent of the Project of Invigorating Health Care through Science, Technology and Education (QNRC2016165), and the Foundation of top notch young and middle-aged medical and health talents in Wuxi (BJ2020079). This work was also supported, in part, by the National Basic Research Program of China (973 Program) (No. 2010CB530004).

## Conflict of Interest

The authors declare that the research was conducted in the absence of any commercial or financial relationships that could be construed as a potential conflict of interest.

## Supplementary Material

Supplementary Table 1 Primer sequences used for qRT-PCR.

Supplementary Figure 1 Representative flow cytometry plots of NK cell purity before and after magnetic cell sorting. NK cells were purified from brain mononuclear cells and splenic lymphocytes using a magnetic cell sorting system (MACS) incorporating anti-mice CD49 MicroBeads. The purity of CD3^-^CD49^+^ NK cells in brain and spleen after sorting was over 90% detected by FCM. sNK; NK cells in spleen; bNK, NK cells in brain.

Supplementary Figure 2 Gating strategy for the mouse CD45^+^CD3^-^CD49b^+^ NK cell population by FCM. **(A)** NK cells in spleen of uninfected mice. **(B)** NK cells in spleen of infected mice. **(C)** NK cells in brain of infected mice. Firstly, R1 was gated according to cell size presented by FSC and SSC. Next, CD45^+^ cells in R1 were gated as R2 representing lymphocytes. Then, CD3^-^CD49b^+^ cells in R2 were gated as R3 and identified as the mouse NK cell population. sNK; NK cells in spleen; bNK, NK cells in brain.

Supplementary Figure 3 Construction of a mouse model of *A. cantonensis* infection. Mice were infected with 20 *A. cantonensis* third-stage larvae by intragastric administration and detected at 0, 10, 14, 18 and 22 dpi, respectively. **(A, B)** Survival rate and body weight of mice at different infection time points. **(C)** Representative images of infected mice with neurologic deficit symptoms including erect hair, arched back, falling to one side when walking, blindness. **(D–F)** Neurological impairment scores of infected mice evaluated by Longa’s score, Clark’s general score and Clark’s focal score. **(G)** Representative images of brain tissue in infected mice. The brains tissue of infected mice displayed obvious hemorrhage and fourth-stage larvae (blue arrow) from 14 to 22 dpi. **(H–N)** Expression of inflammatory cytokines IL-1β, IL-6 and TNF-α in brain at different infection time points detected by qRT-PCR and ELISA. Data are expressed as the means ± SD. Data shown represent analysis from two independent experiments with four to twelve mice per group. Survival curve comparison was determined by Log-rank Test. Comparison of body weight was carried by independent-samples T test. Comparison of the neurological impairment scores was compared by non-parametric test. Multiple comparisons of gene and protein levels of cytokines at different infection time-points were performed using one-way ANOVA. **P* < 0.05; ***P* < 0.01; ****P* < 0.001.

Supplementary Figure 4 The percentage and absolute number of splenic T cells in *A. cantonensis*-infected mice. **(A)** The percentage T cells in splenic lymphocytes. **(B)** The absolute number of splenic T cells. Splenic T cells were identified as CD3^+^CD49b^-^ cells and detected by FCM. Data are expressed as the means ± SD. Data shown represent analysis from two independent experiments with three mice per group. Multiple comparisons of the percentage and count of T cells at different time-points of infection were performed by one-way ANOVA. **P* < 0.05; ***P* < 0.01, compared with that of 0 dpi.

Supplementary Figure 5 Representative dot plots stained with isotype controls of anti-NKG2D-PE **(A)** and anti-NKG2A-PE **(B)**. The cells isolated from the brain and spleen were stained with anti-NKG2D-PE, anti-NKG2A-PE or their isotype controls and detected by FCM.
