# Supplementary figures and images for: CX3CL1 Recruits NK Cells Into the Central Nervous System and Aggravates Brain Injury of Mice Caused by Angiostrongylus cantonensis Infection
Source: Front Cell Infect Microbiol. 2021 May 4;11:672720. doi: 10.3389/fcimb.2021.672720 (PMC8129578; doi:10.3389/fcimb.2021.672720)

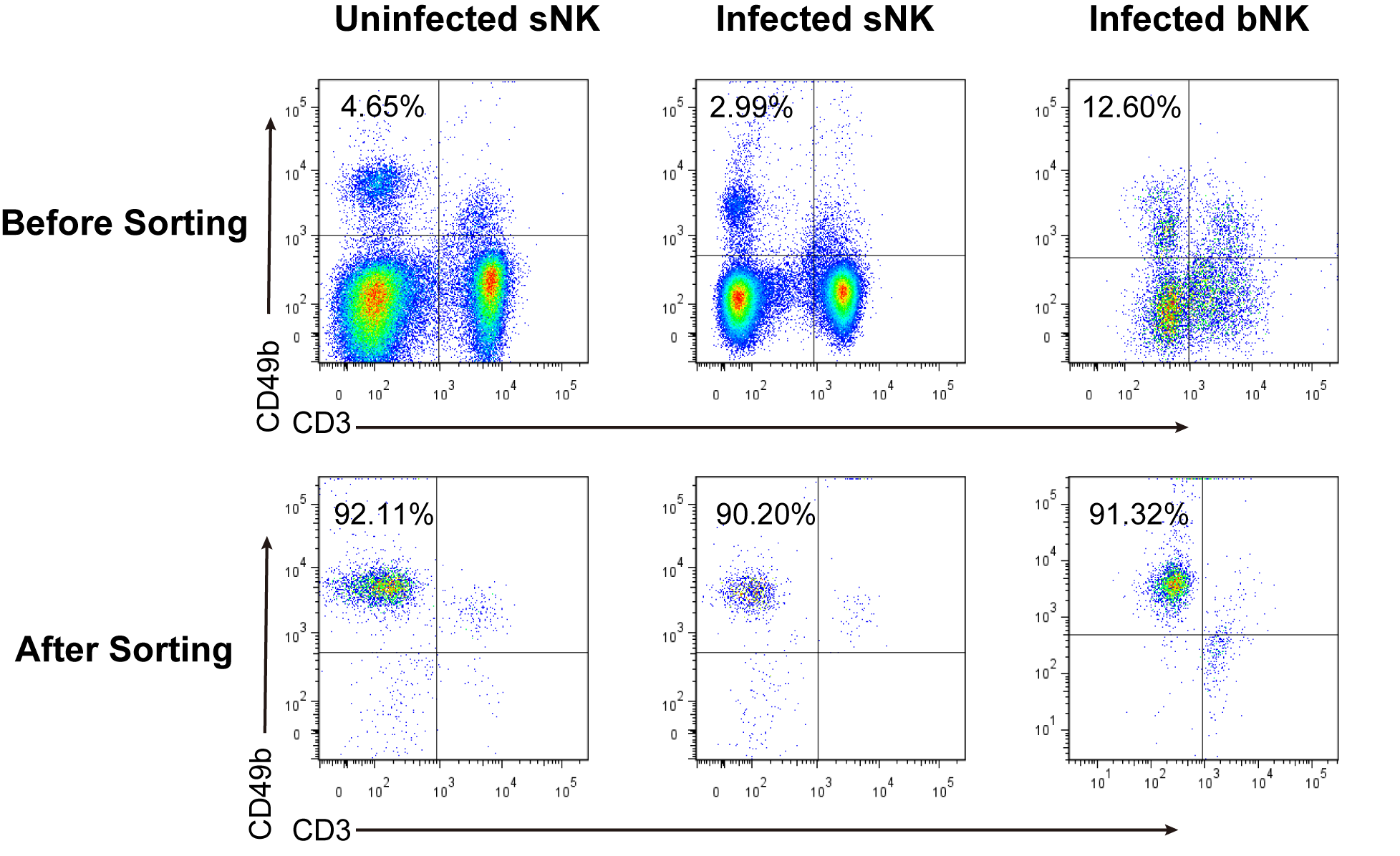

Supplement: Supplementary Figure 1 — Representative flow cytometry plots of NK cell purity before and after magnetic cell sorting. NK cells were purified from brain mononuclear cells and splenic lymphocytes using a magnetic cell sorting system (MACS) incorporating anti-mice CD49 MicroBeads. The purity of CD3-CD49+ NK cells in brain and spleen after sorting was over 90% detected by FCM. sNK; NK cells in spleen; bNK, NK cells in brain. [file Image_1.tif]

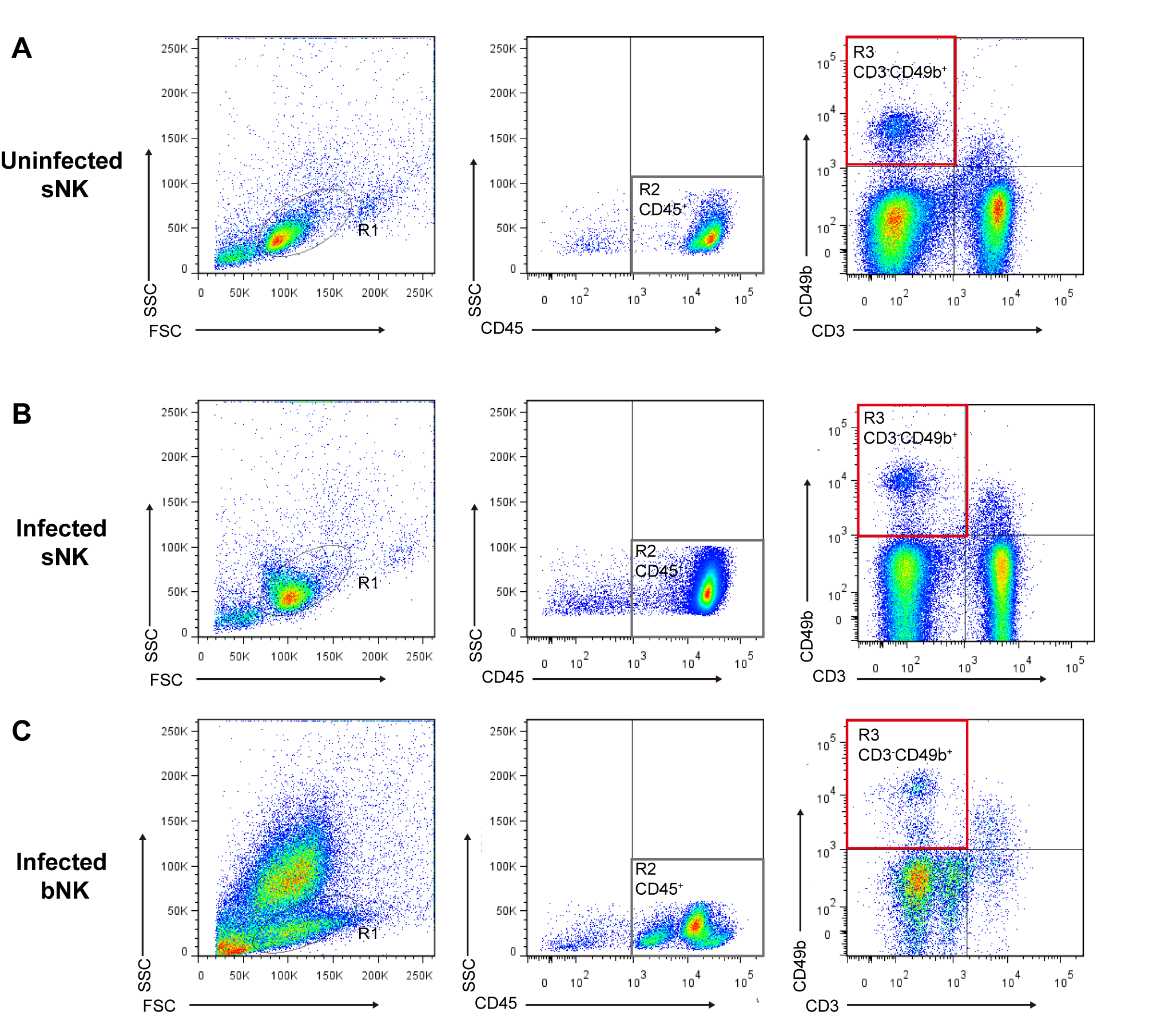

Supplement: Supplementary Figure 2 — Gating strategy for the mouse CD45+CD3-CD49b+ NK cell population by FCM. (A) NK cells in spleen of uninfected mice. (B) NK cells in spleen of infected mice. (C) NK cells in brain of infected mice. Firstly, R1 was gated according to cell size presented by FSC and SSC. Next, CD45+ cells in R1 were gated as R2 representing lymphocytes. Then, CD3-CD49b+ cells in R2 were gated as R3 and identified as the mouse NK cell population. sNK; NK cells in spleen; bNK, NK cells in brain. [file Image_2.tif]

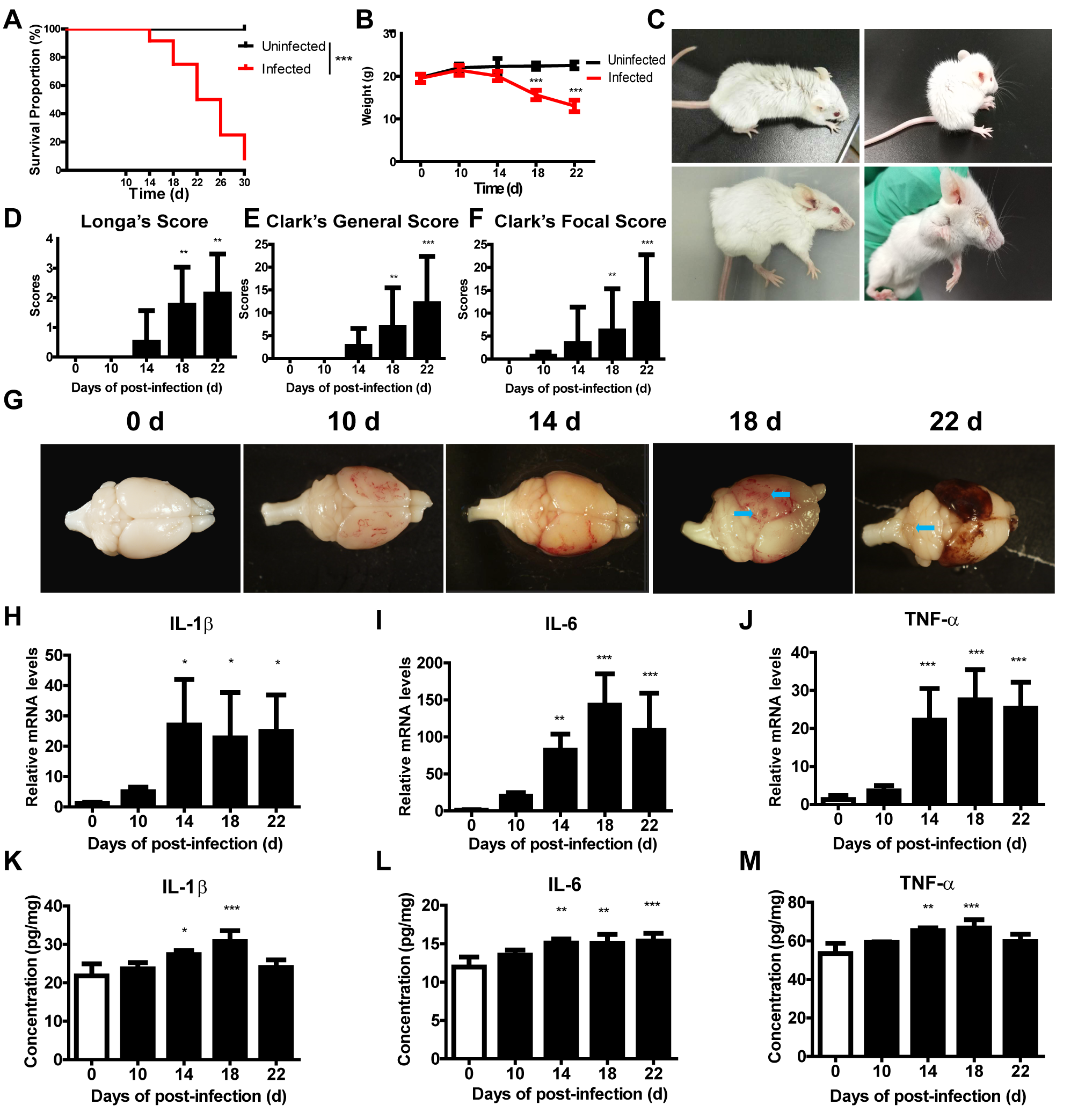

Supplement: Supplementary Figure 3 — Construction of a mouse model of A. cantonensis infection. Mice were infected with 20 A. cantonensis third-stage larvae by intragastric administration and detected at 0, 10, 14, 18 and 22 dpi, respectively. (A, B) Survival rate and body weight of mice at different infection time points. (C) Representative images of infected mice with neurologic deficit symptoms including erect hair, arched back, falling to one side when walking, blindness. (D–F) Neurological impairment scores of infected mice evaluated by Longa’s score, Clark’s general score and Clark’s focal score. (G) Representative images of brain tissue in infected mice. The brains tissue of infected mice displayed obvious hemorrhage and fourth-stage larvae (blue arrow) from 14 to 22 dpi. (H–N) Expression of inflammatory cytokines IL-1β, IL-6 and TNF-α in brain at different infection time points detected by qRT-PCR and ELISA. Data are expressed as the means ± SD. Data shown represent analysis from two independent experiments with four to twelve mice per group. Survival curve comparison was determined by Log-rank Test. Comparison of body weight was carried by independent-samples T test. Comparison of the neurological impairment scores was compared by non-parametric test. Multiple comparisons of gene and protein levels of cytokines at different infection time-points were performed using one-way ANOVA. *P < 0.05; **P < 0.01; ***P < 0.001. [file Image_3.tif]

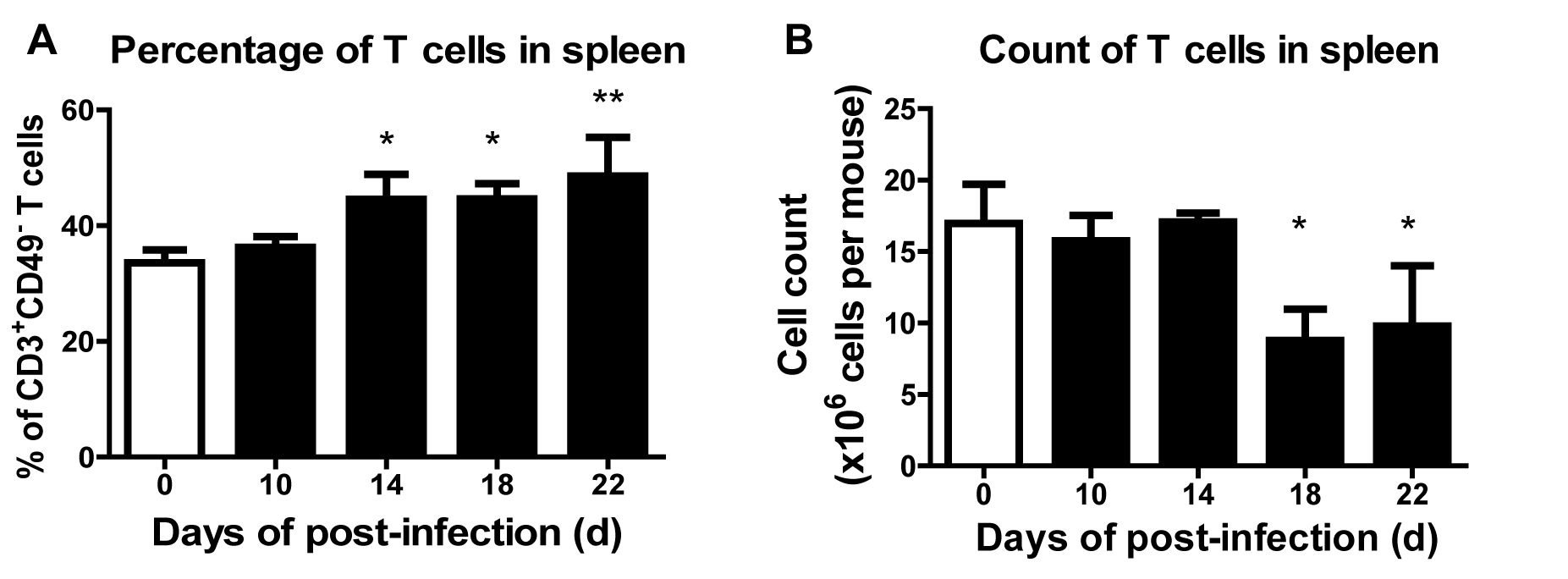

Supplement: Supplementary Figure 4 — The percentage and absolute number of splenic T cells in A. cantonensis-infected mice. (A) The percentage T cells in splenic lymphocytes. (B) The absolute number of splenic T cells. Splenic T cells were identified as CD3+CD49b- cells and detected by FCM. Data are expressed as the means ± SD. Data shown represent analysis from two independent experiments with three mice per group. Multiple comparisons of the percentage and count of T cells at different time-points of infection were performed by one-way ANOVA. *P < 0.05; **P < 0.01, compared with that of 0 dpi. [file Image_4.tif]

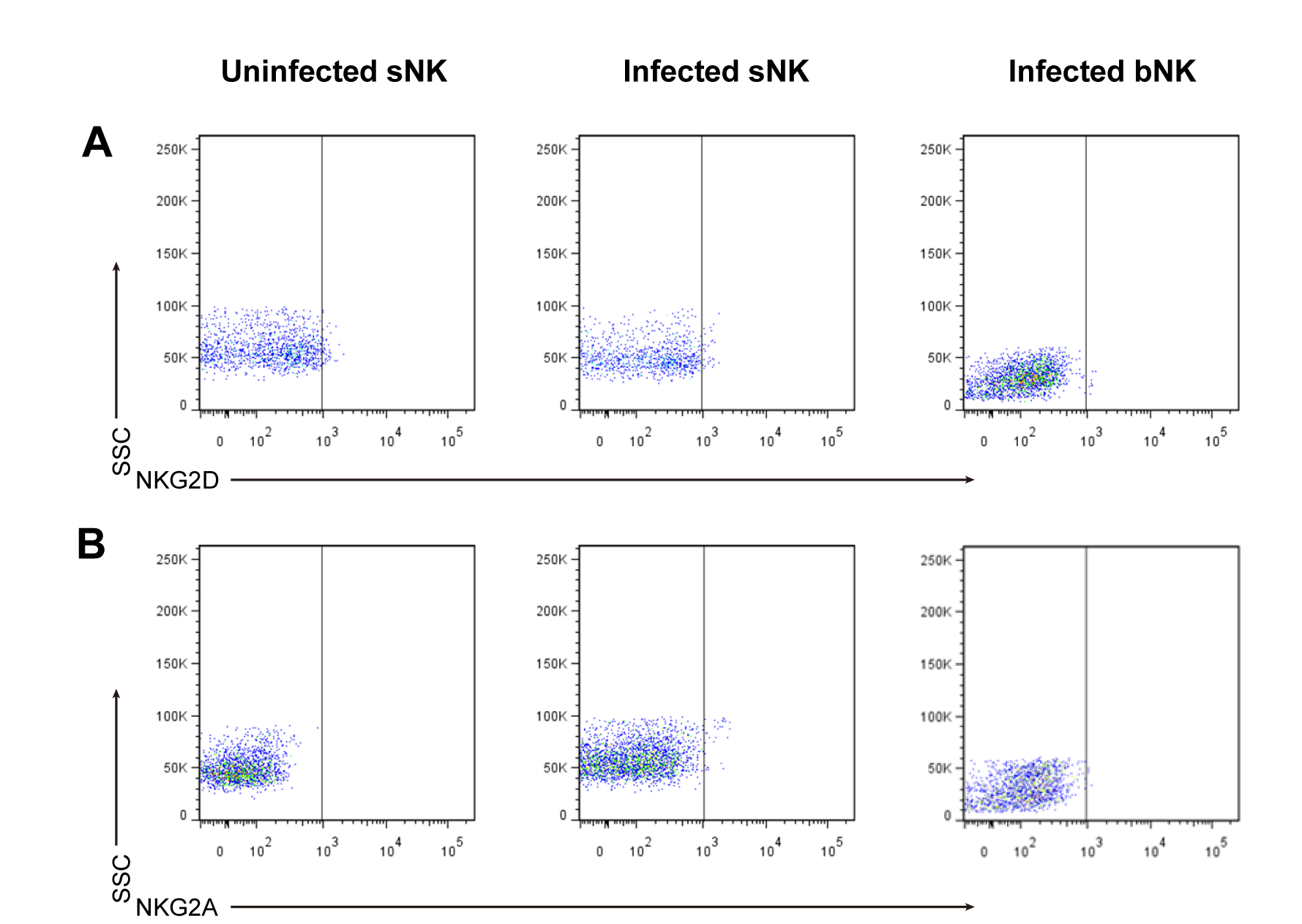

Supplement: Supplementary Figure 5 — Representative dot plots stained with isotype controls of anti-NKG2D-PE (A) and anti-NKG2A-PE (B). The cells isolated from the brain and spleen were stained with anti-NKG2D-PE, anti-NKG2A-PE or their isotype controls and detected by FCM. [file Image_5.tif]
